# A Comparative Study of Forehead Temperature and Core Body Temperature under Varying Ambient Temperature Conditions

**DOI:** 10.3390/ijerph192315883

**Published:** 2022-11-29

**Authors:** Anming Chen, Jia Zhu, Qunxiong Lin, Weiqiang Liu

**Affiliations:** 1 Tsinghua Shenzhen International Graduate School, Tsinghua University, Shenzhen 518055, China; 2 Biomechanics and Biotechnology Lab, Research Institute of Tsinghua University in Shenzhen, Shenzhen 518057, China; 3 Department of Mechanical Engineering, Tsinghua University, Beijing 100084, China; 4 Guangdong Public Security Science and Technology Collaborative Innovation Center, Guangdong Provincial Public Security Department, Guangzhou 510050, China

**Keywords:** forehead temperature, axillary temperature, oral temperature, correlation, agreement

## Abstract

When the ambient temperature, in which a person is situated, fluctuates, the body’s surface temperature will alter proportionally. However, the body’s core temperature will remain relatively steady. Consequently, using body surface temperature to characterize the core body temperature of the human body in varied situations is still highly inaccurate. This research aims to investigate and establish the link between human body surface temperature and core body temperature in a variety of ambient conditions, as well as the associated conversion curves. Methods: Plan an experiment to measure temperature over a thousand times in order to get the corresponding data for human forehead, axillary, and oral temperatures at varying ambient temperatures (14–32 °C). Utilize the axillary and oral temperatures as the core body temperature standards or the control group to investigate the new approach’s accuracy, sensitivity, and specificity for detecting fever/non-fever conditions and the forehead temperature as the experimental group. Analyze the statistical connection, data correlation, and agreement between the forehead temperature and the core body temperature. Results: A total of 1080 tests measuring body temperature were conducted on healthy adults. The average axillary temperature was (36.7 ± 0.41) °C, the average oral temperature was (36.7 ± 0.33) °C, and the average forehead temperature was (36.2 ± 0.30) °C as a result of the shift in ambient temperature. The forehead temperature was 0.5 °C lower than the average of the axillary and oral temperatures. The Pearson correlation coefficient between axillary and oral temperatures was 0.41 (95% CI, 0.28–0.52), between axillary and forehead temperatures was 0.07 (95% CI, −0.07–0.22), and between oral and forehead temperatures was 0.26 (95% CI, 0.11–0.39). The mean differences between the axillary temperature and the oral temperature, the oral temperature and the forehead temperature, and the axillary temperature and the forehead temperature were −0.08 °C, 0.49 °C, and 0.42 °C, respectively, according to a Bland-Altman analysis. Finally, the regression analysis revealed that there was a linear association between the axillary temperature and the forehead temperature, as well as the oral temperature and the forehead temperature due to the change in ambient temperature. Conclusion: The changes in ambient temperature have a substantial impact on the temperature of the forehead. There are significant differences between the forehead and axillary temperatures, as well as the forehead and oral temperatures, when the ambient temperature is low. As the ambient temperature rises, the forehead temperature tends to progressively converge with the axillary and oral temperatures. In clinical or daily applications, it is not advised to utilize the forehead temperature derived from an uncorrected infrared thermometer as the foundation for a body temperature screening in public venues such as hospital outpatient clinics, shopping malls, airports, and train stations.

## 1. Introduction

As one of the most prevalent illness detection tools, the monitoring of body temperature serves an essential role in the screening of diseases with fever symptoms as a precursor [1]. The ideal human body temperature detection system should be able to correctly and rapidly acquire and present the core temperature of the human body with simple, convenient, and straightforward operation, steady accuracy, and little external impact [2]. Currently, the armpit, oral cavity, tympanic cavity, rectum, forehead, temporal area, bladder, esophagus, etc. are frequently the locations for measuring body temperature [3,4,5]. In everyday body temperature detection or clinical applications, mercury thermometers are typically employed to capture the axillary or oral temperature as the human body’s normal temperature, while there are relatively few temperature observations in the bladder and esophagus [6,7]. A rectal temperature measurement is the gold standard for clinical temperature monitoring in children, but it is rarely utilized in adults [8,9]. With the advancement of electronic technology, non-contact methods for measuring body temperature are progressively gaining popular acceptance, and the applications for measuring temperature in the tympanic cavity, temporal area, and forehead are increasing [10].

The global spread of the new coronavirus pandemic is becoming increasingly severe, and fever is a common symptom in the early onset of the vast majority of new coronavirus infections. Therefore, the detection of body temperature has become an essential method for the prevention and control of epidemics in various occasions at home and abroad [11,12]. Rapid, efficient, and non-contact body temperature detection and screening needs have accelerated the spread of non-contact body temperature measuring and forehead temperature detection techniques. Currently, handheld infrared thermal imagers are commonly used as body temperature screening equipment in a restricted number of crowds, such as residential neighborhoods or highway entrances. This type of equipment is characterized by its small size, high precision, low price, portability, and ease of use. Fixed infrared thermal imagers are frequently used as body temperature screening equipment in a large variety of mobile population situations, such as airports, stations, subways, and large commercial centers. During the pandemic, infrared temperature thermal imagers have become the standard method for the rapid screening of individual body temperatures [13,14,15].

The infrared thermal imager detects the surface temperatures of the human body, including the forehead and temporal temperatures. Affected by factors such as ambient temperature, the measured result may not accurately represent the human body’s core temperature, which is usually represented by the axillary temperature, oral temperature, and rectal temperature. While the body surface temperature measured in a cold environment is rather low, the core body temperature remains steady and not necessarily low. In relatively warm tropical regions, the recorded body surface temperature is relatively high, but the core body temperature is not necessarily elevated [16,17]. In addition, the majority of thermal imagers currently in use are often modified from industrial thermal imagers. Although they offer the benefits of high accuracy and the capacity to do multi-target simultaneous dynamic monitoring, they have a number of limitations. When used for medical purposes to screen a large number of people for an abnormal body temperature, the measurement data has a large dispersion due to the interference from factors such as different application temperatures in different regions and different distances between fevered individuals and the temperature measuring probe [18]. Daniel et al. [19] also discovered that for the same forehead surface temperature recorded by an electronic thermometer, the temperature variation between several detections was substantial. They concluded that this discrepancy might lead to erroneous and deceptive disease monitoring data. An independent verification is necessary prior to the application. Therefore, it is vital to establish the device offset and error compensation of the ambient temperature for the infrared thermal imager in order to guarantee that it retains accurate and efficient detection capabilities in varying temperature environments and crowded areas. This remains one of the most pressing concerns that needs immediate attention.

The body temperature that is displayed by infrared thermal imagers is essentially a two-dimensional projection of the human body temperature. The device of the infrared thermal imager is cumbersome and costly to use. It is mostly used to identify aberrant temperature increases in a specific body site, as opposed to individual body temperature increases as a sign of illness. Clinically, infrared thermal imagers are frequently used for the screening and early detection of cancers, such as thyroid cancer [20] and breast cancer [21]. In densely populated areas, this body temperature measurement is not utilized. To actualize this application, several researchers at home and abroad have conducted extensive studies. Goh et al. [22] noted that the accuracy of infrared thermometers is impacted by factors such as the tester’s distance and the forehead measuring location and accomplished a degree of error correction by modifying and configuring the devices. Xu et al. [23] attempted to use the multiple linear regression fitting algorithm to correct the influence of ambient temperatures on the measurement results of body temperature. However, this algorithm is only applicable when the fluctuation of the factors influencing body temperature is small, and it cannot produce accurate results when the range of ambient temperature changes is large. Some scholars have proposed installing a heating device controlled by a proportional-integral-derivative algorithm and pulse width modulation technology on the sensor of an infrared thermometer to compensate for the thermal shock effect. However, there has been no research on ambient temperature compensation [24]. Some researchers have devised a dual-band infrared thermometer to overcome the problem of recalibration when the ambient temperature varies by taking into account the impact of the radiation factors inside the tested person and the equipment [25]. In the specific design and use of infrared thermometers, although many studies have designed solutions for its current limitations, the current error compensation of infrared thermal imagers for ambient temperature is still an incomplete problem. Furthermore, there is no detailed and accurate research report on the correlation between the forehead temperature and the core body temperature.

Therefore, acquiring the surface temperature of the human body using an infrared thermal imager and immediately displaying the core body temperature of the human body still requires a huge number of scientific tests. The purpose of this study is to determine the relationship between the human body surface temperature represented by the forehead temperature and the core body temperature represented by the axillary temperature and the oral temperature. We compare the trend of changes between the body surface temperature and the core body temperature, such that the forehead temperature may directly reflect the core body temperature in different ambient temperatures. In future practical applications, forehead temperature detection finally enables the rapid screening of people with an abnormal body temperature in the event of a large crowd flow.

## 2. Materials and Methods

### 2.1. Ethics Statement

The human forehead temperature and core body temperature were collected from all volunteers who were recruited from society or schools at a certain hourly wage. They were informed about the study, data confidentiality, relevance of the research, and signed an informed consent form before the study started. The collection of the human forehead temperature and core body temperature data was approved by the local ethics committee at Tsinghua University (Approval number: 2022-F104).

### 2.2. Study Design

Three well-recognized temperature measurement instruments, including a mercury thermometer, an electronic thermometer, and a thermal imager, were selected for this study. We recruited six healthy participants between the ages of 23 and 32 (independent of gender) and performed a total of 1080 temperature tests in the armpit, oral cavity, and forehead, obtaining the relevant data of the armpit, oral cavity and forehead temperatures at various ambient temperatures (14 °C to 32 °C). The axillary and oral temperatures were employed as the control group, and the forehead temperature was employed as the experimental group.

As previously stated, we can use the armpit, oral cavity, tympanic cavity, rectum, forehead, temporal area, bladder, and esophagus to detect the temperature of the human body at this stage. However, which locations of body temperature can best represent the core body temperature? Pecoraro et al. [3] provided a summary of the research on detecting body temperature in hospital systems using electronic thermometers, infrared thermometers, and mercury thermometers. According to the adopted research, hospital researchers most commonly chose rectal temperature as the reference value, which was sufficient to demonstrate the validity of rectal temperature as the core body temperature. Concurrently, numerous researchers chose the axillary temperature, oral temperature, or temporal temperature as the reference value. In addition, Umiska et al. [4] found that monitoring the temperature of the esophagus and bladder simultaneously in the control of mild therapeutic hypothermia is a more accurate way to reflect the change in the human body’s core temperature. It can be seen that the rectal or esophageal temperature is commonly recognized as a reliable alternative value of core body temperature at present. Due to the specificity of the measured region and professional standards, this core body temperature measurement is mostly utilized in pediatrics, emergency departments, and operating rooms, which have stringent requirements for the accuracy of human body temperature measurements. Kanegaye et al. [26] found that the underarm temperature is an insensitive indicator of fever that defines the resistance to treatment and suggested that the body temperature should be measured by an oral or rectal route for an adjudication of the treatment resistance in Kawasaki Disease. Nevertheless, Mazerolle et al. [27] reported that the oral temperature was unsuitable as the core temperature due to the influence of external temperature, placement position, and fluid intake. Affected by certain factors in some application situations, the axillary temperature or the oral temperature may not accurately reflect the core body temperature. However, the axillary temperature or the oral temperature still continues to be chosen to represent the core body temperature after comprehensively considering the reliability, recognition, and feasibility of the data acquisition. Specifically, it is employed as a reference value in the screening of ordinary fever personnel and numerous application situations where the temperature requirement is not particularly stringent, as well as in the investigation of some hospital scenarios [28,29].

### 2.3. Experimental Equipments

The selection and final determination of all the instruments utilized in this study were in accordance with and fulfilled the requirements of the standard ISO 80601-2-56:2017 medical electrical equipment—part 2-56: the particular requirements for basic safety and essential performance of clinical thermometers for body temperature measurement. Before beginning the data collecting experiment, the experimental operator calibrated all the equipment in accordance with the manufacturer’s instructions, and all equipment was stored as necessary, dedicated to this study, and not utilized for any other purpose.

All the experimental equipment and related parameters used in our experiment are as follows: (1) a Yuyue mercury thermometer (Jiangsu Yuyue Medical Equipment Co., Ltd., Shanghai, China)—the body temperature was measured at the armpit, measurement range = 35–42 °C, error range = ±0.1 °C; (2) a Yuyue electronic thermometer YT318 (Jiangsu Yuyue Medical Equipment Co., Ltd., Shanghai, China)—the body temperature was measured in the oral cavity, measurement range = 32–42.9 °C, error range = ±0.1 °C; (3) a Hikvision handheld temperature measurement thermal imager TBC-3117-3/U (Hangzhou Hikvision Digital Technology Co., Ltd., Hangzhou, China)—the body temperature was measured on the forehead center, the highest level of calibration accuracy = ±0.5 °C, measurement range = 25–50 °C; (4) an ESPEC constant temperature chamber GLW-160/1 (Guangzhou ESPEC Environmental Instrument Co., Ltd., Guangzhou China)—size 1900 × 3200 × 2600mm (depth × width × height), temperature range = −40–80 °C, humidity range = 20–95% RH, temperature fluctuation ≤ ±0.5 °C. The chamber was loaded for this investigation while the operator and all of the recruited subjects were inside. At a low temperature, all the subjects inside the chamber generated and released heat into the chamber space. At a high temperature, they absorbed the heat created by the chamber. All of these processes had some effects on the chamber’s temperature stability. In order to minimize the impact of potential loading effects, the chamber was utilized in precise conformity with the test procedure regulations outlined in this experiment.

Initialization is a crucial procedure that must be highlighted for all the experimental equipment in this study. After each measurement and prior to the next measurement, all the thermometers were reset. The mercury thermometer was initiated, and the digital thermometer and thermal imager were restarted. The preceding procedure aimed to ensure the consistency of each measurement’s starting point and reduce the impact of any uncertain system components on the measurement results.

### 2.4. Preparations

To ensure the reliability and repeatability of the experimental results, some preparations were done prior to the start of the experiment. First, there subjects avoided intense activity for 30 min before the measurement, as well as eating, drinking, and applying cold or hot compresses. Second, the availability of all equipment was checked. For the mercury thermometers and electronic thermometers, if the thermometer’s inaccuracy surpassed ±0.2 °C, the mercury column fell on its own, or the glass tube contained cracks when the measuring end was placed into the constant temperature warm water with a measured temperature between 38 °C and 40 °C, the thermometer was unqualified and could not be used. For an infrared temperature measurement thermal imager, it was necessary to ensure that the sweat on the subject’s forehead was dry, that the hair had been removed, and that the thermometer was aimed at the subject’s forehead just 2 cm above the center of the eyebrows, which is in a position close to vertical.

### 2.5. Measurement Methods

As ordinary thermometers, mercury thermometers and electronic thermometers are generally simple to use. Due to the particular requirements of the test duration for the electronic thermometers and the test process settings in this study, the test time for both thermometers reached 7 min.

The measurement method of the forehead temperature needs to be explained in detail in this study. During a mercury thermometer measurement, an infrared temperature measurement thermal imager is utilized to simultaneously measure the same subject. It has been studied that the skin temperature of the different measurement points on the face varies greatly, among which the forehead skin temperature is the highest [30]. The external measured surface temperature is mainly a value reflected by the convection and radiant heat exchange between the human skin and the surrounding air [31]. The uncertainty of the skin reflectivity and some other factors will affect this process, which will lead to an inhomogeneous distribution of our measured surface temperature, such as the forehead region’s inhomogeneous temperature distribution. Some scholars have studied the difference of the emissivity between the different skin tones. The latest research shows that there is no difference in the thermal emissivity between black and white skin, and there is no significant difference in the influence of skin pigmentation on skin reflectivity [32,33,34]. Therefore, while conducting the forehead temperature experiments, it is unnecessary to account for the variation in skin reflectivity induced by skin color. The key is to establish the consistency of each measurement’s position and distance, so as to ensure the relative stability of the external conditions of convection and radiant heat exchange between the human skin and the surrounding air during each measurement. In this study, the specifications for measuring the position and distance are as follows: the same operator must ensure that the thermometer is positioned 2 cm above the center of the subject’s forehead and eyebrows, at a distance of 15 cm. The data are then read and recorded, and the value is rounded to two decimal places. The operator must ensure that each measurement condition is consistent in order to minimize the errors caused by the uncertainty in skin reflectivity.

### 2.6. Measurement Process

The measuring procedure in this investigation followed a strict operating sequence. 

First, body temperature data were measured in the morning (from 8:30 to 12:00) and in the afternoon (from 13:00 to 18:00) for a total of 2 days. Each tester held a mercury thermometer and an oral thermometer. After the ambient temperature and humidity of the constant temperature chamber attained the preset temperature after 10 min, both the operator and the six subjects entered the chamber and waited for 20 to 30 min in this setting before the test began. During the duration of waiting, all the subjects remained standing. Additionally, during the subsequent temperature test, the subject maintained a standing position while the temperature was taken and recorded.

Second, each participant simultaneously inserted a preprepared and calibrated electronic thermometer into the chosen area of the oral cavity and began the timing. 

Third, each participant placed a mercury thermometer in the specified region under the armpit immediately after placing the oral electronic thermometer. There was a 5–10 s delay. The value of the axillary temperature tended to be constant after 5 min of measurement, with no statistically significant difference between the consecutive variations over time, while it took seven minutes for the oral temperature to stabilize. Therefore, the mercury thermometer under the armpit was inserted last and removed first to guarantee that the oral temperature test period was rigorously maintained at seven minutes to assure test accuracy.

Fourth, the central forehead temperature was monitored by an infrared thermal imager at 3 min, 5 min, and 7 min during the mercury thermometer test. 

Fifth, all the subjects repeated the preceding measurement procedure six times in a row under the same ambient temperature point and varied time intervals.

Sixth, the operator adjusted the temperature of the constant temperature chamber to the next temperature point after completing the test of the first temperature point. After about 20 min, the constant temperature chamber reached the newly set temperature point. Then, all the subjects repeated steps 1 to 5 to carry out the body temperature test at the new temperature point. The test time of one temperature point was about 2.5 h.

### 2.7. Statistics

A statistical analysis was performed using SPSS 26.0 (IBM SPSS Statistics Software, New York, NY, USA) and MedCalc version 20.022 (MedCalc Software, Mariakerke, Belgium) statistical software. The descriptive analyses of measurement data were expressed as mean ± standard deviation, where the standard deviation was the statistical component of the uncertainty. The paired *t*-test was used to compare the mean temperature readings, and the one-way ANOVA test was used to compare the level of statistical significance between the mean temperature readings at various temperature points. The Pearson correlation coefficient was determined to assess the correlation between the axillary temperature, oral temperature, and forehead temperature values that were obtained from an infrared thermal imager, which was consistent with numerous previous studies of a similar nature and considered statistically significant at *p* < 0.001. Analyses of Bland-Altman plots were conducted to evaluate the agreement of the measurements across multiple devices, and *p* < 0.05 was deemed statistically significant. The regression analysis package was utilized for the curve prediction and linear curve fitting [35,36,37].

## 3. Results

### 3.1. Overall Comparison of Body Temperature Data

During the experiment, 1080 temperature tests were repeated on three distinct body sections of healthy individuals aged 23 to 32 using three distinct measuring instruments to get the axillary temperature, oral temperature, and forehead temperature under different environmental conditions. The values obtained for the axillary temperature, oral temperature, and forehead temperature are summarized in Table 1. All the data indicated that the average axillary temperature was (36.7 ± 0.41) °C, the average oral temperature was (36.7 ± 0.33) °C, and the average forehead temperature was (36.2 ± 0.30) °C. Using the paired *t*-tests, it was determined that the average axillary and oral temperatures were significantly higher than the average forehead temperatures (*p* = 0.000 and *p* = 0.000, respectively).

**Figure 1:**
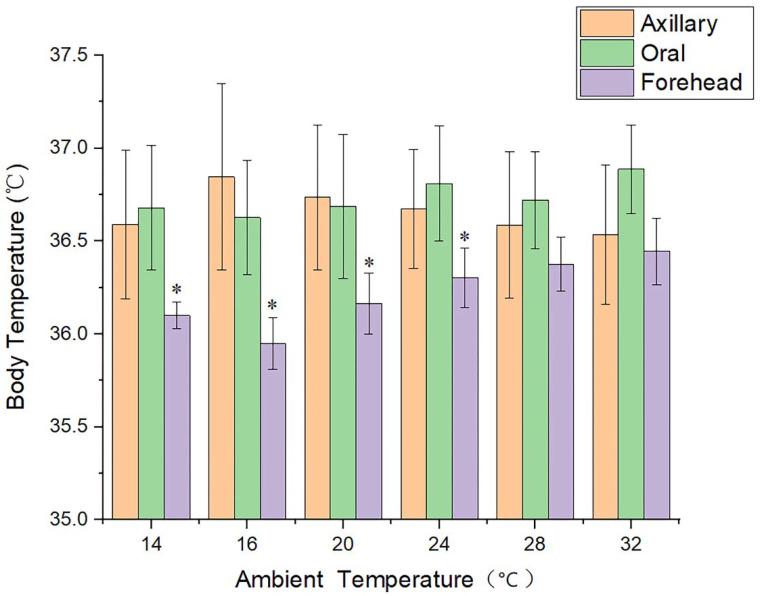
Histogram of the axillary temperature, oral temperature, and forehead temperature data under different ambient temperature conditions. * means significant statistical difference.

When the ambient temperature was 14 °C, 16 °C, 20 °C, or 24 °C, a one-way ANOVA test revealed statistically significant differences (*p* < 0.05) between the forehead temperature and the axillary temperature, as well as the forehead temperature and the oral temperature, but not between the axillary temperature and the oral temperature. At temperatures of 28 °C and 32 °C, there was no statistically significant difference between the axillary, oral, and forehead temperatures. Figure 1 displays the data distribution and significant differences for the axillary temperature, oral temperature, and forehead temperature.

### 3.2. Correlations 

**Figure 2:**
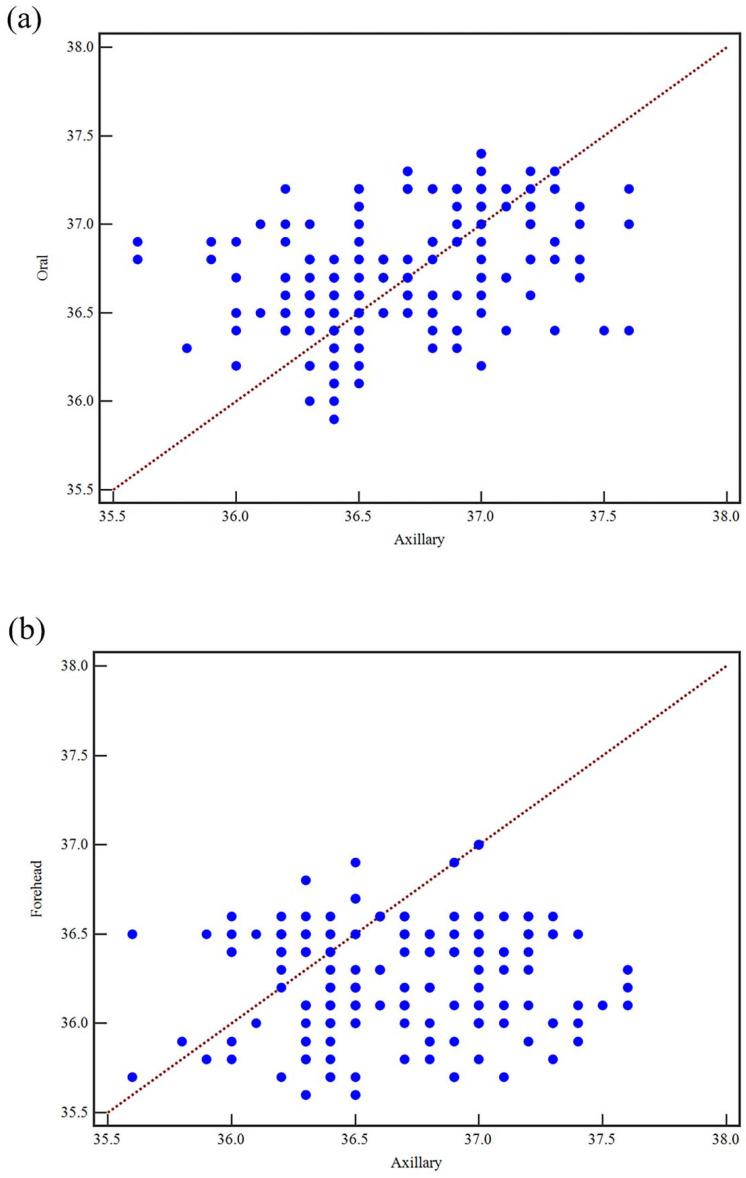

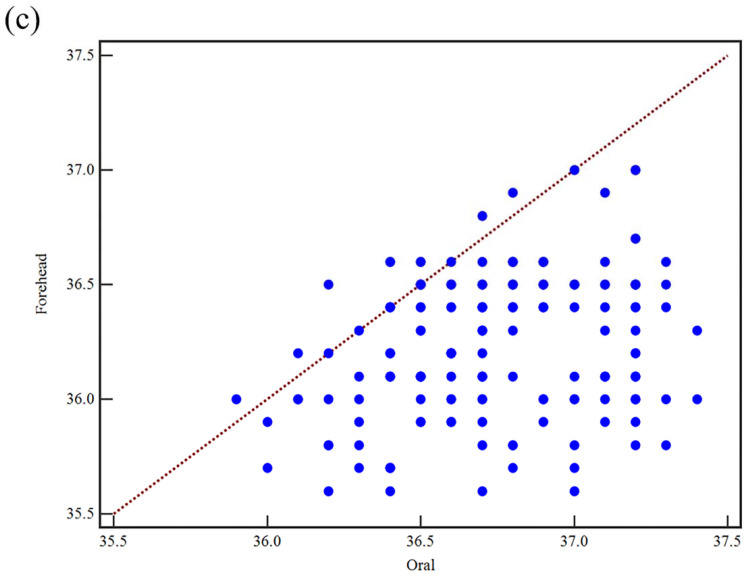
Scatter diagram of the correlation between the axillary temperature, oral temperature and forehead temperature; (**a**) correlation between the axillary temperature and oral temperature data (r = 0.41; *p* < 0.001; 95% CI, 0.28–0.52), (**b**) correlation between the axillary and forehead temperature data (r = 0.07; *p* = 0.33; 95% CI, −0.07–0.22), (**c**) correlation between the oral and forehead temperature data (r = 0.26; *p* < 0.001; 95% CI, 0.11–0.39).

The correlation coefficient between the axillary temperature and the oral temperature was 0.41 (*p* < 0.001; 95% CI: 0.28–0.52), indicating a moderate positive correlation that was statistically significant. When evaluating and comparing the forehead temperature data with other data, a very weak positive correlation was revealed between the forehead temperature and the axillary temperature, with a correlation value of 0.07 (*p* = 0.33; 95% CI, −0.07–0.22). There was a small but statistically significant correlation between the forehead temperature and the oral temperature, with a correlation coefficient of 0.26 (*p* < 0.001; 95% CI, 0.11–0.39). Figure 2 depicts the correlation scatter plots between the axillary and oral temperatures, the axillary and forehead temperatures, and the oral and forehead temperatures.

### 3.3. Agreement Evaluations

**Figure 3:**
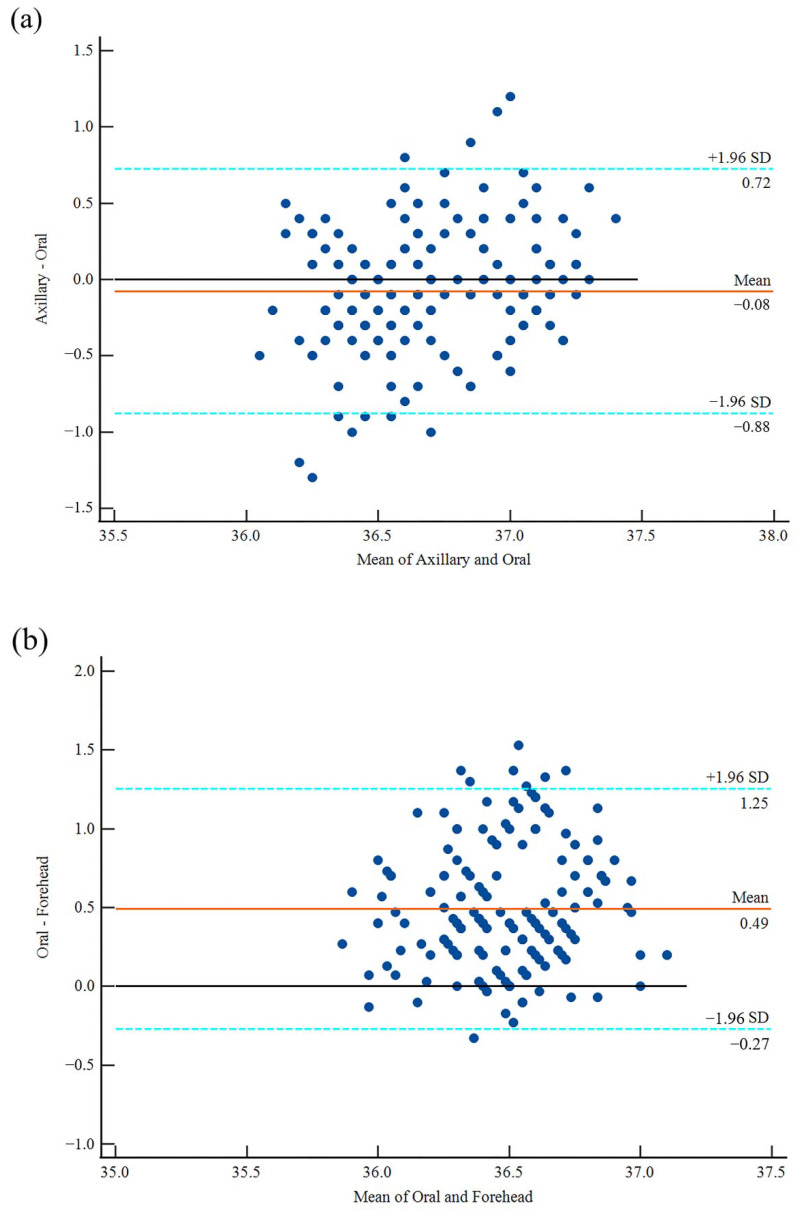

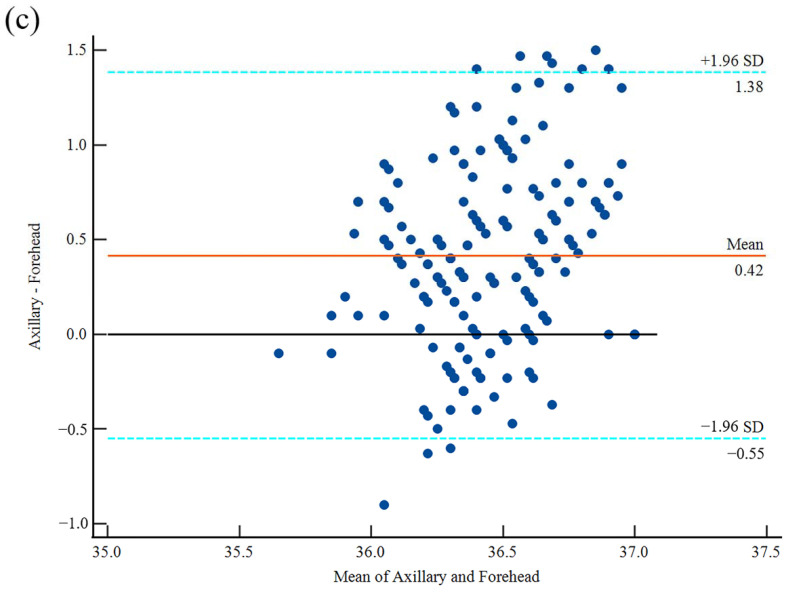
Bland–Altman plot with the mean difference and 95% limits of agreement; (**a**) the axillary and oral temperature data, (**b**) the oral temperature and forehead temperature data, (**c**) the axillary temperature and forehead temperature data.

The mean difference between the axillary temperature and the oral temperature was −0.08 ± 0.41 °C. The 95% confidence interval of the agreement limits for the axillary and the oral temperatures ranged from −0.88 °C to 0.72 °C. The Bland-Altman figure revealed that the majority of the data points were densely packed around the zero line that represented the difference between the two temperature readings, with just 4.4% (8/180) of the values surpassing the 95% level of confidence (Figure 3a).

The mean difference between the mean oral temperature and forehead temperature was 0.49 ± 0.39 °C. The 95% confidence interval of the agreement limits for the axillary and the oral temperatures was between −0.27 °C and 1.25 °C. As shown in Figure 3b, the Bland-Altman plot showed that most of the data points were tightly clustered around the zero line of the difference between the two temperature readings, with just 4.4% (8/180) of the values surpassing the 95% level of confidence.

The mean difference between the mean values of the axillary and the forehead temperatures was 0.42 ± 0.49 °C. The 95% confidence interval of the agreement limits for the axillary and oral temperatures ranged from −0.55 °C to 1.38 °C. According to the Bland-Altman plot, the bulk of the data points were tightly packed around the zero line that indicated the difference between the two temperature values, with 5.6% (10/180) of the values exceeding the 95% level of confidence (Figure 3c).

### 3.4. Regression Analysis

There was a linear association between the axillary temperature and the forehead temperature, as well as the oral temperature and the forehead temperature, as determined using the curve estimation feature of the data analysis program SPSS regression analysis. In this study, the bivariate linear regression was used with the axillary or the oral temperature as the dependent variable, and the ambient temperature and the forehead temperature as independent variables, and fitting with a 95% confidence interval to obtain the fitting equation f (x, y) = a × x + b × y + c. The relevant parameters are shown in Table 2 below.

## 4. Discussion

In order to comprehensively examine the effect of the ambient temperature variations on the human body surface temperature, this study utilized the most typical temperature range in public settings, which ranged from 14 °C to 32 °C. Six incremental temperature points of 14 °C, 16 °C, 20 °C, 24 °C, 28 °C, and 32 °C were chosen as the constant ambient temperature for the collection of the experimental data. This study first compared the average axillary, oral, and forehead temperatures, and then analyzed the correlation between the three body temperature measurements. The Bland-Altman analysis was then utilized to determine the level of agreement between the three independent variables. Finally, the association between the forehead temperature, axillary temperature, and oral temperature was identified based on the results of the preceding comparative analysis, and the linear regression curves between the forehead temperature, axillary temperature, and oral temperature were fitted using regression analysis.

There are currently few studies on the influence of changes in the ambient temperature on the human body temperature, and more studies are being conducted on the accuracy or validity of body temperature data collected by different human body temperature monitoring techniques at a certain ambient temperature. Dolibog et al. [38] discovered that the axillary temperature measured by a contact thermometer was higher than the forehead temperature obtained by a non-contact thermometer, and that the body temperature data acquired by the two techniques had a high level of statistical reliability. Tang et al. [39] conducted a systematic meta-analysis evaluating the efficacy of several measures. The analysis revealed that there was no statistical difference between the forehead temperature measured by an infrared thermometer and the oral temperature measured by a mercury thermometer. However, there was a statistically significant difference between the forehead temperature and the axillary temperature measured by a mercury thermometer. In this study, we discovered that the axillary temperature and the oral temperature did not vary significantly in response to changes in the external environmental temperature. Rather, the axillary temperature and the oral temperature fluctuated around 36.7 °C, with no discernible upward or downward trend in the data. There was no statistically significant difference between the two, which demonstrated the validity of the axillary temperature and the oral temperature as the standard reference body temperatures of healthy individuals, in accordance with the current clinical application results [40,41,42].

Regarding the non-contact infrared measurement of body surface temperature, several researchers have conducted relevant studies. Zaproudin et al. [43] evaluated the repeatability of infrared thermometers that are used to detect body surface temperature by employing an intra-class correlation analysis. The average intra-class correlation (ICC) analysis level was 0.88, demonstrating that the non-contact infrared thermometer had high repeatability in monitoring body surface temperature. Packham et al. [44] demonstrated the accuracy of non-contact infrared thermometers for measuring body surface temperature by employing an assessment approach similar to Zaproudin et al. [43]. Chen et al. [45] investigated the time efficiency of non-contact infrared thermometers in measuring body temperature using recovered hospitalized patients as research subjects. They concluded that this method was more time efficient than using axillary mercury thermometers and infrared tympanic thermometers and could effectively prevent unnecessary patient suffering. Our investigation revealed that under the conditions of fluctuating ambient temperature, the statistical value of the forehead temperature exhibited a clear upward trend with increasing ambient temperature, which was significantly influenced by the fluctuating ambient temperature, though the forehead temperature was slightly higher at 14 °C than at 16 °C. The explanation could be the measuring system’s uncertainty, that is, the systematic component of the uncertainty, which we could only limit as much as possible but could not fully prevent.

The results of this study indicate that the average axillary and oral temperatures are within the widely accepted normal temperature range for healthy individuals. However, the average forehead temperature is somewhat below this range [46,47,48]. As shown in Figure 1, the forehead temperature is considerably impacted by the changes in ambient temperature, and has a statistically significant difference with the axillary temperature or the oral temperature, especially when the ambient temperature is low. As the temperature rises, the differential level lowers progressively. When the ambient temperature exceeds 28 °C, the differences between the three become insignificant. It can be seen that when the ambient temperature reaches a specific level, the forehead temperature data acquired by the non-contact infrared thermal imager is close to the core body temperature, suggesting that it may reflect the core body temperature. When the ambient temperature is below this value, there is a significant difference between the forehead temperature and the core body temperature. Currently, it is factually inaccurate to use forehead temperature as an indicator of the core body temperature, and it has no reference value for clinical diagnosis or public body temperature monitoring. Furthermore, the conclusions of Niven et al. [49] confirmed the aforementioned findings of our investigation.

Numerous investigations on the correlation between the forehead temperature and the axillary temperature or the oral temperature have demonstrated that the correlation is reasonably strong in an enclosed setting where the ambient temperature is generally steady. In a somewhat steady indoor setting of 24–26 °C, Apa et al. [50] discovered a substantial and significant positive association between the axillary temperature and the forehead temperature, with a correlation value of 0.76. Fortuna et al. [51] compared the forehead data measured by a non-contact infrared thermometer with body temperature data obtained by a rectal thermometer in a conventional room temperature environment. The results demonstrated a significant strong correlation between the two, with a correlation coefficient of 0.952. Obviously, a strong correlation does not imply that the two approaches are interchangeable in every way. High correlations will also result from constant differences between the two approaches, which are unacceptable in clinical practices. [52,53,54].

This study investigated the correlation between the body temperature data acquired from three distinct temperature measurement techniques at varying ambient temperatures. In the presence of continuously changing ambient temperature factors, we discovered a significant moderate positive correlation between the axillary temperature and the oral temperature, almost no correlation between the axillary temperature and the forehead temperature, and a significant weak correlation between the oral temperature and the forehead temperature, which differed somewhat from the conclusions of previous studies [55,56,57]. The results from this study’s preliminary analysis suggested that when the ambient temperature changes, the correlation between the axillary temperature and the oral temperature, which represents the core body temperature, remains stable, whereas the correlation between the forehead temperature and the core body temperature is greatly affected, with either no correlation or a weak correlation. Therefore, when utilizing a non-contact infrared thermal imager to measure the forehead temperature to define the core body temperature, it is vital to account for the changes in ambient temperature components in their entirety. Hausfater et al. [58] achieved similar conclusions to ours, namely that there was a weak connection between the forehead temperature obtained by non-contact infrared thermometers and the tympanic membrane measurement representing the core body temperature. The ambient temperature had a substantial impact on the forehead temperature that was obtained by this method. In addition, the tendency of overestimating body temperatures with low values and underestimating body temperatures with high values also emerged. They suggested that the forehead temperature readings from infrared thermometers could not provide a credible basis for the screening of fever outpatients.

Using the Bland-Altman analysis approach, a large number of researchers have investigated the agreement of body temperature data derived from diverse measuring techniques. Senser et al. [59] examined the agreement of a non-contact infrared thermometer, an infrared tympanic thermometer, and an electronic axillary thermometer in detecting body temperature in a group of adult emergency department patients. It was claimed that there was a lack of agreement between the body temperature data gathered by the aforementioned three techniques in the adult emergency department population, and that the non-contact infrared thermometer should be employed in the medical sector with greater agreement limits. Blake et al. [60] investigated the agreement of non-contact infrared thermometers and disposable oral electronic devices for detecting body temperature in outpatients with comparable findings. However, the results indicated that the degree of the agreement did not match the criteria. The use of non-contact infrared thermometers as a diagnostic criterion in afebrile outpatients was deemed unsuitable. Nonetheless, several researchers have reached the conclusion that the agreement is satisfactory. Wang et al. [32] tested the forehead temperature with three distinct forehead thermometers, and the findings were in good agreement with the mercury thermometer data. Chiappini et al. [61] similarly stated that the forehead temperature and the axillary temperature were in good agreement for the detection of children’s body temperature, and that the non-contact infrared thermometer functioned well.

As depicted in Figure 3a of the Bland-Altman plot, the mean difference between the axillary temperature and the oral temperature does not deviate significantly from the zero line, and only 4.4% of the points fall outside the 95% confidence range, demonstrating a small systematic error and a good agreement between the two techniques. Meanwhile, the mean difference between the forehead temperature and the axillary temperature (Figure 3b) and the mean difference between the forehead temperature and the oral temperature (Figure 3c) are significantly different. Even though only 4.4% of the temperature difference points fall beyond the 95% confidence interval, the mean difference between the forehead and oral temperatures deviates from the zero line by 0.49 °C. All of these indications suggest that the forehead temperature is inconsistent with the axillary and oral temperatures. The findings of this investigation are comparable to those of Senser [59] and Blake et al. [60]. Therefore, it is necessary to analyze the difference between the forehead temperature and the axillary temperature or oral temperature, which represents the core body temperature, and to use a linear regression analysis to fit the relationship between them and ensure that the forehead temperature can accurately represent the core body temperature.

Through the overall comparison, correlation comparison, and Bland-Altman plot comparison analysis, we can determine that under different ambient temperatures, there is no significant difference between the axillary temperature and the oral temperature, there is a relatively high significant positive correlation, the mean difference between the two is close to the zero line, and both values can accurately represent the core body temperature. However, the values for the forehead temperature and the axillary temperature, as well as the forehead temperature and the oral temperature, differ significantly. They exhibit considerable differences and irrelevance at a variety of temperature thresholds. Therefore, the error compensation between the forehead temperature and the actual body temperature must be accomplished before the forehead temperature readings acquired under varying ambient temperatures may be recognized as clinically relevant. Similarly, Mallallah et al. [62] demonstrated that it is necessary to establish a compensation relationship between the human body surface temperature and the core temperature in order to ensure the accuracy of temperature measurements and a minimum mean absolute error between the forehead temperature and the core body temperature.

To verify the validity of the fitted curve, we selected all the forehead temperature data that was acquired under various ambient temperatures and inserted them into the above fitting equation to obtain the predicted value using the same number of samples as the measured values of the axillary temperature and the oral temperature. Then, the predicted value and the measured value were subjected to a one-way ANOVA significance level test. This study indicated that there was no significant difference between the fitted predicted values and measured values of the axillary temperature and the oral temperature (*p* < 0.05), and the levels of significant difference were 0.969 and 0.797, especially the level of the axillary temperature which was extremely near to 1. The particular characteristics are detailed in Table 3. The comparison of the preceding data confirmed that the predicted value of the linear regression equation and the measured value of the test were roughly comparable. The validity of the curve fitted by linear regression in this study could well be demonstrated by the congruence between the fitting findings and the aforementioned analytical outcomes.

There are still a few limitations in this research. For instance, the ambient temperature range is not that sufficient. To properly illustrate the connection between the fluctuation of the forehead temperature and the variation of the ambient temperature, additional temperature research should be conducted. The preferred minimum temperature range limit is −10 °C for the purpose of simulating the outdoor ambient temperature in winter. The highest limit of the temperature range should ideally be 42 °C to simulate the hot temperature environment in summer. In addition, the sample size must be increased in order to further eliminate research mistakes caused by individual variances in the samples. If sufficient subjects are available, the testing of different age groups can be enhanced so that the difference between the forehead temperature and the core body temperature can be analyzed more precisely, and different error compensation ranges can be established for different groups of individuals. In the end, it is assured that the forehead temperature represents the core body temperature more accurately. Although there is still a great deal of work to be supplemented and perfected in the study of using the forehead temperature to represent the core body temperature of the human body, this study provides favorable data support and method support for this topic and is one of the key links for future advancement in this direction. Consequently, this research has significant application value and importance.

## 5. Conclusions

This study investigated the numerical differences, correlations, and agreements between the forehead, axillary, and oral temperatures taken at various ambient temperatures. The quantitative transformation linkage between the two components of the ambient temperature and the forehead and core body temperature represented by the axillary temperature and the oral temperature was established. On the basis of our investigation and findings, we conclude that the fluctuations in the ambient temperature have a significant impact on the forehead temperature. There are significant differences between the forehead and axillary temperatures, as well as the forehead and oral temperatures, when the ambient temperature is low. As the ambient temperature rises, the forehead temperature tends to progressively converge with the axillary and oral temperatures. In summary, it is not recommended to use the forehead temperature obtained by an infrared thermal imager without the error compensation as the body temperature screening standard for people in public places, such as hospital outpatient clinics, shopping malls, airports, and stations, particularly as a screening standard for fever. However, the error compensation is affected by numerous characteristics, including not only the ambient temperature, but also the age, sex, and temperature condition of the individual being examined. Therefore, there is currently no unified perspective on the research of the error compensation value. This study mainly analyzes the influence of the ambient temperature on the error compensation. It is considered that the ambient temperature factors should be fully considered to implement the application in these scenarios. The error compensation of the forehead temperature data should be first performed in conjunction with the linear compensation curve between the forehead temperature and the core body temperature, represented by the axillary temperature and the oral temperature, under varying ambient temperatures. Then, the acquired forehead temperature can be regarded as a reference for the core body temperature, but it is also necessary to make a choice after comprehensive consideration according to the temperature requirements of the actual application scenarios.

## Figures and Tables

**Table 1:** Body temperature measurement results with the different measurement methods under different temperature conditions.

Cases	Axillary Temperature (Mean ± SD)/°C	Oral Temperature (Mean ± SD)/°C	Forehead Temperature (Mean ± SD)/°C
14 °C	36.6 ± 0.40	36.7 ± 0.33	36.1 ± 0.07 *
16 °C	36.8 ± 0.50	36.6 ± 0.31	36.0 ± 0.14 *
20 °C	36.7 ± 0.39	36.7 ± 0.39	36.2 ± 0.16 *
24 °C	36.7 ± 0.32	36.8 ± 0.31	36.3 ± 0.16 *
28 °C	36.6 ± 0.39	36.7 ± 0.26	36.4 ± 0.15
32 °C	36.5 ± 0.38	36.9 ± 0.24	36.5 ± 0.18
Average	36.7 ± 0.41	36.7 ± 0.33	36.2 ± 0.30 *

Note: * *p* < 0.05.

**Table 2:** Linear regression fitting parameter table of the forehead temperature, axillary temperature and oral temperature.

Model	AT			OT		
	Value	SD	*p*	Value	SD	*p*
C—c	26.335	4.272	0.000	29.093	3.424	0.000
FT—a	0.296	0.120	0.015	0.207	0.096	0.032
A-T—b	−0.018	0.006	0.002	0.006	0.005	0.228

Note: C: constant, AT: axillary temperature, OT: oral temperature, FT: forehead temperature, A-T: ambient temperature, SD: standard deviation p: significance level.

**Table 3:** Comparative analysis of the predicted value of the regression analysis curve and the measured value of the experiment.

Group	Category	Mean ± SD (°C)	Range (°C)	*p*
AT	Predicted Value	36.66 ± 0.10	36.44–36.96	0.969
	Measured Value	36.7 ± 0.41	35.6–37.6	
OT	Predicted Value	36.74 ± 0.09	36.56–36.93	0.797
	Measured Value	36.7 ± 0.33	35.9–37.4	

Note: AT: axillary temperature, OT: oral temperature, *p*: one-way ANOVA significance level.

## Data Availability

Not applicable.
